# Caffeine: What Is Its Role in Pain Medicine?

**DOI:** 10.7759/cureus.25603

**Published:** 2022-06-02

**Authors:** Sri Harsha Boppana, Michael Peterson, Austin L Du, L V Simhachalam Kutikuppala, Rodney A Gabriel

**Affiliations:** 1 Anaesthesiology, University of California, San Diego, La Jolla, USA; 2 Biology, University of California, San Diego, La Jolla, USA; 3 Anesthesiology, University of California, San Diego, La Jolla, USA; 4 Medicine, Konaseema Institute of Medical Sciences & Research Foundation, Amalapuram, IND

**Keywords:** psychoactive drug, pain management, nociception, caffeine, adjuvant therapy, adenosine

## Abstract

Caffeine is the world's most widely used psychoactive legal substance. The involvement of caffeine in pain management has gotten minimal attention in the past, but it is getting more attention now. This article provides a brief assessment of the literature to clarify the role of caffeine as a pain reliever and stimulate the interest of researchers. Caffeine affects adenosine receptors, which are involved in nociception, and plays a significant role in pain regulation. Caffeine's usage as an adjuvant therapy has been extensively documented in the literature, and it is now accessible in certain over-the-counter drugs. The mixture of coffee and morphine for pain reduction in individuals with terminal cancer has shown mixed outcomes in studies. Caffeine can be utilized for hypnic headaches and post-dural puncture headaches since it is crucial in pain regulation. Caffeine has the potential to help in pain management. Caffeine's usage for migraines and end-stage cancer disease is not well acknowledged. Further research is essential to focus on caffeine's potential role in various forms of pain, including dosage escalation and outcome assessment standardization.

## Introduction and background

Caffeine is an alkaloid with the chemical structure "3,7-dihydro-1,3,7-trimethyl-1H-purine-2,6-dione". Caffeine is a methylxanthine-class psychoactive and central nervous system stimulant that is legal throughout the world, apart from the other psychoactive substances [1]. According to historical archives, caffeine's effects were first recognized by a 'goat herder' named 'Khaldi' in southern Abyssinia in 850. However, it was extracted for the first time in 1819 by a German scientist named "Friedlieb Ferdinand Runge" [2,3]. Caffeine-containing beverages such as tea and coffee were popular in Arabian nations in the 15th and 16th centuries and Europe in the 18th and 19th centuries. Caffeine's medicinal effects have been understood long before it became a common ingredient in everyday beverages [4]. Caffeine appears to pose minor health hazards, but its long-term advantages outweigh any potential unfavorable short-term health impacts. Caffeine use in moderation is safe for most healthy, non-pregnant individuals. It boosts alertness, energy, response speed, wakefulness, and the capacity to concentrate and focus attention while also reducing weariness. It also improves physical stamina, short-term memory, and cognitive abilities [5,6]. The involvement of caffeine in pain management is one of the areas that has received minimal attention in the past. This review article aims to provide the reader with a quick assessment of the literature to clarify the role of caffeine as a pain adjunct and to spark the interest of researchers.

## Review

Potential mechanism of caffeine as an analgesic

Pain management has been examined in various circumstances and is constantly being researched [7-10]. As an adjunct in pain treatment, caffeine's role is controversial as more large-scaled high-quality studies are needed to demonstrate its efficacy. Caffeine's effects on pain regulation are complicated [11]. Adenosine inhibits neuronal activity in the 'Central Nervous System (CNS) and Peripheral Nervous System (PNS)'. Adenosine receptors are divided into four subtypes in human bodies: A1, A2A, A2B, and A3, expressed in distinct areas of the CNS and PNS. Adenosine, an endogenous molecule, has a variety of modulatory actions in the central and peripheral nervous systems, and its receptors have been linked to antinociception. Arousal, concentration, and vigilance could all be improved by enhancing these receptors. In neuropathic pain, nociceptive, and inflammatory models, activation of A1 and A2A receptors results in antinociception [12-14]. Caffeine's structure is similar to that of adenosine; hence it competes with it for A2a receptors, leading them to be inhibited. Also, caffeine does not affect dopamine release and does not have the same potential for misuse as other adenosine blockers like cocaine. Caffeine's actions on adenosine receptors may help people feel less discomfort [14-18]. It directly affects pain signaling by inhibiting brain adenosine receptors and peripheral adenosine receptors on sensory afferents. Caffeine's antinociceptive and adjuvant effects may be explained by adenosine receptor antagonistic effects and suppression of cyclooxygenase activity at particular locations [18-20]. Some experts believe that heredity might affect how people react to coffee [21].

Caffeine and its role as an adjuvant component

An 'adjuvant component' is anything given to medication to make it more efficacious [22]. Many over-the-counter (OTC) analgesics contain low dosages of caffeine as an adjuvant in conjunction with antidepressants, acetaminophen, and non-steroidal anti-inflammatory medications. For decades, clinical research has evaluated and proved its adjuvant analgesic benefits [23,24]. It has been suggested that its inhibitory effects on A2A and A2B receptors and inhibition of cyclooxygenase activity are responsible for these effects [11]. When compared to the analgesic alone, adding modest quantities of caffeine to a typical dose of popular analgesics improves pain relief in a small but significant proportion of individuals, according to certain studies. According to a systematic study conducted by Derry et al., the impact mentioned above was minor but statistically significant [22,25]. Derry et al. discovered 19 trials that added 100-130 milligrams of caffeine to the primary medicine, such as ibuprofen or paracetamol, with 7238 individuals. The most common illnesses studied were headaches, postoperative dental pain, and postpartum discomfort. There were no major side effects recorded in these investigations, and the authors indicated that adding 100 milligrams or more of caffeine to an analgesic might be beneficial [22,25].

Regardless of what has been claimed, combining caffeine with morphine has various effects, including inhibition or amplification, depending on the condition and the dosage and method of administration [11]. In a double-blind, randomized, placebo-controlled experiment, researchers evaluated the effectiveness of 200 milligrams of intravenous caffeine infusion once a day for two days as an adjuvant to opioids for pain management in advanced cancer patients. They divided the patients into two groups, one with caffeine and the other with a placebo. They found that while caffeine infusion decreased pain and tiredness, the effect was not clinically significant in patients with advanced cancer receiving opioid treatment [26].

It should be mentioned that, based on existing information, the use of caffeine-containing analgesics is linked to overuse, headaches, physical dependency, and withdrawal symptoms when abruptly stopped. As a result, it is typically suggested that these substances be used only as needed for acute pain relief [24].

Management of headaches with caffeine

Hypnic headache is an uncommon benign primary headache disease that primarily affects middle-aged individuals at night. Raskin initially characterized it in 1988, and the International Classification of Headache Disorders (ICHD) classified it as the "other main headache" (code 4.5) in 2004 [27,28]. Caffeine appears to be the earliest treatment approach for this type of headache and a preventative drug, according to a literature analysis that only included case reports or smaller open case series. Whether in the form of 'a cup of strong black coffee' or caffeine-containing OTC painkillers, caffeine has been an effective therapeutic choice for this type of headache. Severe reactions to coffee can diagnose hypnic headaches as a pathognomonic clinical trait. However, a randomized clinical trial is still required to verify such claims [29-33].

Post-dural puncture headache (PDPH)' is a common consequence of lumbar puncture and a possible side effect of spinal anesthesia. Although the cause of post-dural puncture headache is unknown, it is thought to be caused by CSF leaking into the epidural space, resulting in altered craniospinal elasticity. PDPH is defined by a reduction in pain in the supine position and an increase in discomfort in the standing position [34,35]. Administration of Caffeine as a PDPH therapy drug was originally performed in 1949, and it may be administered orally, intramuscularly, and intravenously [36]. Caffeine is generally recommended as an initial therapy option for this form of headache. Many studies have been undertaken with various administration methods and dosages and differing success rates [37,38]. A randomized, double-blind clinical investigation conducted by Yucel et al. demonstrated that during the first 90 minutes following spinal anesthesia, 1000 mL of normal saline with 500 milligrams of caffeine sodium benzoate or 1000 mL of normal saline were given. Based on the results, giving intravenous caffeine sodium benzoate before and after spinal anesthesia might reduce PDPH rates [39]. Camann et al. conducted a double-blind, placebo-controlled experiment in which forty postpartum patients with PDPH were given 300 milligrams of oral caffeine. Before and after the medicine was given, pain scores were recorded at 4, 8, and 24 hours later. The study results concluded that oral caffeine could be used to treat PDPH pain [38]. Caffeine causes cerebral artery vasoconstriction by inhibiting adenosine receptors, reducing cerebral blood input and brain blood volume. On the other hand, caffeine increases cerebrospinal fluid production via stimulating sodium-potassium pumps [35,40]. The caffeine administration appears to be a non-invasive alternative that could eliminate the necessity for invasive treatments like epidural blood patches [35].

Caffeine is widely used to treat migraine headaches as adjuvant therapy. Due to recent studies concerning migraine etiology, caffeine has played a more prominent role as a therapy option [35,41]. Caffeine can pass the blood-brain barrier and operate as a vasoconstrictor in the cerebral vascular system. An intravenous injection of 60 mg of caffeine citrate was a safe and well-tolerated abortive option for 61 people suffering from severe migraine headaches in a pilot study done in 2014 [42].

Caffeine for postoperative analgesia

Current literature has investigated the effectiveness of caffeine as a standalone treatment for postoperative pain and other postoperative outcomes. For instance, Karunathilake et al. (2012) sought to determine how caffeine affects postoperative pain following oral surgery. Quantitative sensory testing was used on thirty adult patients before and after oral surgery to measure pain, with both self-reported caffeine consumption and measured caffeine plasma concentrations taken into consideration. The study found that patients whom self-reported caffeine consumption experienced higher pain sensitivity, indicated by higher quantitative sensory testing scores. This same group also experienced a lower pain threshold than those who self-reported no caffeine consumption regarding postoperative pain.

Furthermore, patients with a caffeine plasma concentration > 300ng/mL experienced less postoperative pain than patients with a caffeine plasma concentration < 300 ng/mL, although it is noted that analgesic dosage may have been a factor influencing this result [43]. Though these results were somewhat conflicting, Hambrecht-Wiedbusch et al. (2017) concluded that caffeine alone does have the ability to reduce postoperative pain. The researchers used a rat model to examine how caffeine affected postoperative pain due to sleep loss. Before a surgical incision, the rats were deprived of sleep for 6 hours. It was found that caffeine administered to rats at the beginning of sleep deprivation prevented sleep deprivation-induced increase in postoperative pain [44]. Vlisides et al. (2021) studied intraoperative caffeine's effect on postoperative opioid consumption following laparoscopic surgery. This randomized control trial consisted of 60 patients undergoing laparoscopic colorectal and gastrointestinal surgery, with 30 patients given caffeine and the other 30 given a placebo. The study concluded that caffeine did not reduce early postoperative opioid consumption [45].

Other studies have looked at the effectiveness of caffeine as an adjunct to other analgesics for postoperative pain and other postoperative outcomes. The majority have found that such caffeine combinations can reduce postoperative pain. Samieirad et al. (2017) conducted a randomized controlled trial that looked at the effectiveness of caffeine-containing analgesics and codeine-containing analgesics in treating postoperative pain and swelling following implant surgery. Eighty patients undergoing implant surgery were divided into two groups of 40, with one group receiving acetaminophen caffeine capsules and the other group receiving acetaminophen codeine capsules. The study found that codeine-containing analgesics more effectively reduced postoperative pain than caffeine-containing analgesics, while caffeine-containing analgesics more effectively reduced swelling. It was concluded that caffeine-containing analgesics effectively reduce postoperative pain and swelling [46]. Forbes et al. (1991) conducted a clinical trial that studied caffeine's effect on ibuprofen analgesia regarding postoperative pain from oral surgery.

Two hundred ninety-eight outpatients reporting postoperative pain from oral surgery were randomly assigned ibuprofen, different concentrations of ibuprofen and caffeine, or a placebo. The study concluded that the ibuprofen and caffeine treatment was 2.4 - 2.8 times more potent than an ibuprofen monotherapy. Furthermore, the analgesic action of the ibuprofen and caffeine treatment lasted longer and had a faster onset of postoperative pain following oral surgery [47]. Forbes et al. (1990) conducted another clinical trial that studied the monotherapeutic effects of aspirin, caffeine, and the combination of aspirin and caffeine in postoperative pain following oral surgery. Three hundred fifty outpatients were given either aspirin, caffeine, a combination of aspirin and caffeine, or a placebo. The study concluded that every treatment except caffeine alone significantly reduced postoperative pain compared to the placebo. A combination of aspirin and caffeine reduced postoperative pain in patients with a severe baseline pain than an aspirin monotherapy [48]. McQuay et al. (1996) completed a clinical trial that compared the efficacy of an ibuprofen monotherapy with a combination of ibuprofen and caffeine in treating postoperative pain from oral surgery. One hundred sixty-one patients who underwent oral surgery were given either ibuprofen or a combination of ibuprofen and caffeine. The study found that adding caffeine to ibuprofen increased the onset of ibuprofen's effect and was therefore superior in reducing postoperative pain following oral surgery compared to an ibuprofen monotherapy [49]. Weiser et al. (2018) organized a similar clinical trial that compared the effects of a combination drug containing ibuprofen and caffeine with ibuprofen alone, caffeine alone, and a placebo. A baseline pain intensity was measured for 748 patients, 562 treated with one of the various treatment options listed previously. The study found that a combination of ibuprofen and caffeine was better in reducing postoperative pain following oral surgery than ibuprofen alone [50].

Unlike the previous studies, Po et al. (1998) did not find the same significant relationship between a caffeine adjunct and postoperative pain. A meta-analysis was conducted to determine the efficacy of an ibuprofen monotherapy compared to ibuprofen and codeine or ibuprofen and caffeine on postoperative pain. The study concluded that the ibuprofen/codeine combination reduced postoperative pain by 8% and increased the adverse effects of ibuprofen. The ibuprofen/caffeine combination yielded inconsistent results regarding postoperative pain [51].

## Conclusions

Caffeine is extensively consumed in drinks and food across the world, and it has several essential medicinal applications. Its primary function in acute pain therapy is as an adjuvant in the form of fixed combinations with other analgesics. Caffeine is widely used as a stimulant and has effects on multiple systems. Tolerance is suitable for most patients, as one would anticipate over-the-counter medications, and adverse events are nearly always modest and temporary. Involvement of Caffeine in pain management has less attention in the past. The current work conducted a quick assessment of the literature to elucidate caffeine's position as a pain reliever and encourage researchers in this field. When caffeine is administered as an adjuvant, the difference between short and long-acting analgesics, for example, has not been well studied. More high-quality prospective clinical trials are needed better to understand the efficacy of caffeine in various pain conditions.
